# Characteristics associated with COVID-19 or other respiratory viruses’ infections at a single-center emergency department

**DOI:** 10.1371/journal.pone.0243261

**Published:** 2020-12-03

**Authors:** Donia Bouzid, Jimmy Mullaert, Quentin Le Hingrat, Odile Laurent, Xavier Duval, Xavier Lescure, Jean-François Timsit, Diane Descamps, Philippe Montravers, Christophe Choquet, Jean-Christophe Lucet, Enrique Casalino, Benoit Visseaux, ED influenza study group

**Affiliations:** 1 INSERM, IAME, Université de Paris, Paris, France; 2 AP-HP Nord, Emergency Department, Bichat-Claude Bernard University Hospital, Paris, France; 3 AP-HP Nord, Department of Epidemiology, Biostatistics and Clinical Research, Bichat-Claude Bernard University Hospital, Paris, France; 4 AP-HP Nord, Virology Department, Bichat-Claude Bernard University Hospital, Paris, France; 5 AP-HP Nord, Infectious Diseases Department, Bichat-Claude Bernard University Hospital, Paris, France; 6 AP-HP Nord, Medical and Infectious Diseases Intensive Care Unit, Bichat-Claude Bernard University Hospital, Paris, France; 7 AP-HP Nord, Department of Anesthesiology and Critical Care Medicine, Bichat-Claude Bernard University Hospital, Paris, France; 8 AP-HP Nord, Infection Control Unit, Bichat-Claude Bernard University Hospital, Paris, France; University Magna Graecia of Catanzaro, ITALY

## Abstract

**Background:**

Rapid identification of patients with high suspicion of COVID-19 will become a challenge with the co-circulation of multiple respiratory viruses (RVs). We have identified clinical or biological characteristics to help distinguish SARS-CoV-2 from other RVs.

**Methods:**

We used a prospective cohort including all consecutive patients admitted through the emergency department’s (ED) and presenting respiratory symptoms from November 2019 to April 2020. Patients were tested for RV using multiplex polymerase chain reaction (mPCR) and SARS-CoV-2 RT-PCR.

**Results:**

203/508 patients were positive for an RV during the non-SARS-CoV-2 epidemic period (November to February), and 268/596 patients were SARS-CoV-2 positive during the SARS-CoV-2 epidemic (March to April). Younger age, male gender, fever, absence of expectoration and absence of chronic lung disease were statistically associated with SARS-CoV-2 detection. Combining these variables allowed for the distinguishing of SARS-CoV-2 infections with 83, 65, 75 and 76% sensitivity, specificity, PPV and NPV, respectively.

**Conclusion:**

Patients’ characteristics associated with a positive PCR are common between SARS-CoV-2 and other RVs, but a simple discrimination of strong SARS-CoV-2 suspicion with a limited set of clinical features seems possible. Such scoring could be useful but has to be prospectively evaluated and will not eliminate the need for rapid PCR assays.

## Introduction

The first cases of coronavirus disease 2019 (COVID-19) were reported in December 2019, in Wuhan, China [1]. After a rapid spread worldwide, the disease was declared a pandemic in March 2020. As of August 10, 2020, over 19 million confirmed cases of COVID-19, including 728,013 deaths [2]. In France, the first case of COVID-19 was identified in Bichat Claude Bernard university hospital, in Paris, on January 24, 2020, concomitant with the end of the annual influenza epidemic [3]. As in most countries struck by the virus, the pandemic's first wave was controlled in France due to a national lockdown that began on March 16, 2020. However, new waves are striking again in several countries with initially controlled outbreaks, and the pandemic is still accelerating worldwide [4].

The virus is thus highly likely to continue to circulate for a prolonged period. In such a scenario, the SARS-CoV-2 virus will likely co-circulate with other respiratory viruses (RVs) during the next fall and winter in the northern hemisphere or during the ongoing months in the southern hemisphere [5]. Such co-circulation was briefly observed in Northern America and Europe at the beginning of the SARS-CoV-2 local outbreaks [6, 7]. As patients presenting with influenza-like illnesses (ILIs) could be infected by any RV, including SARS-CoV-2, this will raise an additional challenge for patients' diagnosis and isolation in an emergency department (ED). The looming threat of concurrent SARS-CoV-2 and other RV epidemics is an increasing concern for both physicians and health policies [8]. Being able to quickly identify and isolate patients who are highly suspected of having COVID-19 is indeed a cornerstone of preventing nosocomial transmission of this new and deadly infection in crowded EDs and other units.

We took advantage of an ongoing prospective study assessing the impact of point-of-care testing with multiplex PCR in our ED to extend the study. We aimed to identify clinical and biological characteristics that help to differentiate COVID-19 from other respiratory viral infections.

## Methods

### Patients and inclusion criteria

Bichat-Claude Bernard hospital is a large 850-bed university reference center for high-risk pathogens in Northern Paris. All consecutive patients presenting with an ILI and admitted to the hospital through the ED were prospectively included from November 18, 2019, to March 30, 2020. All were tested for respiratory viral infection using mPCR assay (see below). Our ED's first COVID-19 case was identified on February 28, and systematic on-site specific SARS-CoV-2 RT-PCR began on March 2, 2020.

An ILI was defined as the association of at least one systemic symptom (fever with a temperature higher than 38.5°, malaise, headache, and myalgia) with one respiratory symptom (cough, sore throat, and dyspnea), according to the eCDC ILI definition [9]. Those indications of PCR testing were identical during both periods in our ED despite the constraints of the epidemics.

### Study periods

In France, the influenza epidemic period extended from November 18, 2019, to March 2, 2020. During this period, SARS-CoV-2 was not spreading in the community or among patients returning or traveling from foreign countries, notably Northern Italy and China. All suspicions of SARS-CoV-2 based on these exposures were thus admitted from other EDs or hospitals in the dedicated ward of the Infectious Diseases department and were not included in this study. Starting on March 3, all patients with an ILI visiting the ED were systematically suspected of COVID-19 and tested for both human RVs and SARS-CoV-2.

To reflect these two periods, they were analyzed separately: (i) Period 1, hereafter called the respiratory virus (RV) period, with an active circulation of non-SARS-CoV-2 RVs, from November 18, 2019 to March 2, 2020, and (ii) Period 2, hereafter called the SARS-CoV-2 period with active circulation of SARS-CoV-2, starting on March 3.

### Virological testing

During the RV period, multiplex PCR was performed as point of care using the QIAstat-Dx Respiratory Panel (QIAGEN, Hilden, Germany). This rapid multiplex PCR assay allows for detecting 22 viral and bacterial respiratory targets, including influenza, parainfluenza, rhinoviruses/enteroviruses, RSV, metapneumovirus, adenovirus, coronaviruses, bocavirus, Mycoplasma pneumoniae, Legionella pneumophila and Bordetella pertussis [10]. During the SARS-CoV-2 period, and depending on kit availability, viral investigations were conducted either with the QIAstat-Dx Respiratory SARS-CoV-2 Panel (Qiagen, Hilden, Germany), allowing for the detection of the same respiratory pathogens plus SARS-CoV-2 [11], or with a combination of the RT-PCR RealStar SARS-CoV-2 Kit RUO (Altona Diagnostics, Hamburg, Germany) [12] and rapid multiplex PCR FilmArray RP2 (BioFire, BioMérieux, Marcy-L'Etoile, France) [13], allowing for the detection of the same viral respiratory pathogens except bocavirus.

### Clinical and biological data

Demographic and clinical data were prospectively collected, including age, sex, respiratory symptoms (e.g., cough, dyspnea, expectoration, chest pain, and auscultatory abnormalities), time since the onset of symptoms, comorbidities (e.g., diabetes, history of stroke, myocardial infarction, chronic heart failure, chronic kidney failure, chronic bronchitis, and asthma) and clinical parameters at ED admission (e.g., temperature, blood pressure, cardiac frequency, respiratory frequency, oxygen saturation, and Glasgow coma score). The following biological data were also recorded: blood counts, C-Reactive Protein, and NT-pro brain natriuretic peptide (NT pro-BNP). Based on the ongoing acquisition of COVID-19-related symptoms, diarrhea, anosmia, and ageusia were added to data recording after the beginning of the SARS-CoV-2 period.

### Modeling strategy for assessing the effect of the co-circulation of SARS-CoV-2 and other respiratory viruses

Under the hypothesis of a co-circulation of SARS-CoV-2 and other RVs, we modelled the odds of having a positive PCR for SARS-CoV-2 with respect to other viruses, given that the PCR is positive. For the sake of clarity, we denoted the sample by S, with the convention S = 1 for the SARS-CoV-2 period and S = 2 for the RV period. We considered a mixture of SARS-CoV-2 and RV period with parameter p = P(S = 1). According to Bayes formula, the probability of a random observation from the mixture actually being drawn from Sample 1 given that the PCR is positive is as follows:

$$P(S=1|PCR+)=\frac{P(PCR+|S=1)p}{P(PCR+|S=1)p+P(PCR+|S=2)(1-p)}$$

and the corresponding odds are

$$O(S=1|PCR+)=\frac{P(PCR+|S=1)p}{P(PCR+|S=2)(1-p)}$$

Therefore, if we consider a binary covariate X, the odds ratio for being COVID+ in the strata X = 1 with respect to the strata X = 0 can be written as follows:

$${OR}_{S,X|PCR+}=\frac{{RR}_{PCR,X|1}}{{RR}_{PCR,X|2}}{OR}_{S,X}$$ (1)

where *RR_PCR,X|s_* denotes the relative risk of having a positive PCR in the strata X = 1 with respect to X = 0, given that the observation belongs to sample *s*. This equality, initially derived in the case of a binary covariate X, can be straight forwardly extended to the case of categorical or quantitative variables and covariate-adjusted quantities.

Eq (1) has a clear interpretation in the sense that it identifies two independent contributions to the quantity *OR_S,X|PCR+_*. The first one is the ratio between two relative risks and reflects the difference of association between the covariate and the PCR diagnosis in both samples. If, for example, the male sex is strongly associated with a higher risk of having a positive PCR for SARS-CoV-2, but no association exists for other RVs, then a positive PCR drawn from the mixture sample tends to more likely be for SARS-CoV-2 if the patient is male. The second contribution comes from the imbalance between both samples for covariate X. If, for example, patients from the SARS-CoV-2 period are significantly younger, then a positive PCR in a young patient is more likely to be a COVID case.

### Statistical analysis

The baseline characteristics within each group were described with numbers and percentages for qualitative variables and median and interquartile range (IQR) for quantitative variables.

We assessed factors associated with a positive PCR for SARS-CoV-2 in the SARS-CoV-2 period and for other RVs in the RV period using univariate logistic regression. For each sample and variable, we reported the Odd ratio (OR), the 95% confidence interval using a normal approximation and the p-value corresponding to the Wald statistic. We used a threshold of 0.05 for statistical significance. We then performed a multivariate model resulting from a stepwise selection procedure among variables with a p-value below 0.05 in the univariate analysis and a proportion of missing values below 20%.

Regarding the modelling of the SARS-CoV-2 risk in a co-circulation period with other RVs, we estimated relative risks on the right-hand side of Eq (1) with a quasi-Poisson regression model to take over-dispersion into account. Since these relative risks vary according to the proportion of positive PCR in both samples, we built different scenarios corresponding to different degrees of prevalence of positive PCR for SARS-CoV-2 and other RVs. Relative risks for these scenarios were estimated with a-priori weights on the observations. The quantity 〖OR〗_(S,X) and its standard error were estimated with a logistic regression from both samples' union. Finally, the estimate for 〖OR〗_(S,X|PCR+) was reported, and its 95% confidence interval was derived with a normal approximation under the hypothesis of independence between its components.

We also performed a multivariate model for the quantity 〖OR〗_(S,X|PCR+) by using multivariate models for the right-hand side of (1) and a stepwise selection procedure based on covariates with p < 0.05 in the univariate analysis and less than 20% of missing data. Moreover, to evaluate the multivariate model's discrimination, we reported the AUC and its 95% confidence interval, derived from 2,000 bootstrap replicates. We finally proposed a clinical score from the multivariate analysis by rounding estimates, and we assessed its discrimination performances (AUC, sensibility and specificity for a given threshold). All analyses were performed using R v4.0.2.

The study has been approved by our local ethics committee of Bichat Claude Bernard hospital (CEERB N2019-050).

## Results

### Patients' demographic characteristics

During the RV and SARS-CoV-2 study periods, 508 and 596 patients were included, respectively. Patients were more frequently male during both periods: 57% (289/508) and 59% (354/596), respectively. The median age was 73 [63–85] years and 60 [46–76] years during the RV and SARS-CoV-2 periods, respectively (p < 0.001). Detailed patients' characteristics are depicted in Table 1.

**Table 1:** Factors associated with a positive PCR for both periods (univariate logistic regression, *p for diff measures statistical significance for the difference between two estimated OR).

		RV period					SARS-CoV-2 period					p for diff*
		Overall (N = 508)	PCR + (N = 203)	PCR—(N = 305)	OR (95%CI) for PCR+	p	Overall (N = 596)	PCR + (N = 268)	PCR—(N = 328)	OR (95%CI) for PCR+	p	
**General**	Male gender	289 (57)	114 (56)	172 (56)	1.0 (0.7–1.4)	0.99	354 (59)	190 (71)	164 (50)	2.4 (1.7–3.4)	**<0.001**	**0.00042**
	Age	73 (61–85)	70 (59–82)	73 (63–85)	1.0 (1.0–1.0)	**0.02**	60 (46–76)	59 (49–73)	62 (43–78)	1.0 (1.0–1.0)	0.54	0.16
	Symptoms duration (days)	2 (1–4)	3 (1–4)	2 (1–4)	1.0 (1.0–1.0)	0.63	3 (2–7)	4 (3–7)	3 (1–7)	1.0 (1.0–1.0)	0.85	0.62
**Symptoms**	Feverishness	208 (41)	102 (50)	105 (34)	2.0 (1.4–2.8)	**<0.001**	373 (63)	215 (80)	158 (48)	4.4 (3.0–6.4)	**<0.001**	**0.0026**
	Hypothermia	21 (4.2)	6 (3)	14 (5)	0.6 (0.2–1.6)	0.36	19 (3)	6 (2)	13 (4)	0.6 (0.2–1.4)	0.24	0.84
	Chills	82 (16)	35 (17)	47 (15)	1.2 (0.7–1.9)	0.54	121 (20)	71 (26)	50 (15)	2.0 (1.3–3.0)	**<0.001**	0.088
	Sweats	38 (7.5)	17 (8)	21 (7)	1.2 (0.6–2.4)	0.52	58 (10)	29 (11)	29 (9)	1.3 (0.7–2.2)	0.42	0.99
	Headaches	28 (5.6)	11 (5)	17 (6)	1.0 (0.4–2.1)	0.95	78 (13)	41 (15)	37 (11)	1.4 (0.9–2.3)	0.15	0.42
	Myalgia	68 (14)	29 (14)	36 (10)	1.3 (0.7–2.1)	0.4	134 (23)	81 (30)	53 (16)	2.2 (1.5–3.3)	**<0.001**	0.082
	Malaise	40 (7.9)	14 (6)	26 (8)	0.8 (0.4–1.6)	0.52	33 (6)	13 (5)	20 (6)	0.8 (0.4–1.6)	0.51	0.97
	Cough	311 (62)	148 (73)	159 (52)	2.6 (1.8–3.9)	**<0.001**	402 (67)	199 (74)	203 (62)	1.8 (1.3–2.5)	**0.0014**	0.16
	Sore Throat	24 (4.8)	12 (6)	11 (4)	1.7 (0.7–4.0)	0.22	41 (7)	21 (8)	20 (6)	1.3 (0.7–2.5)	0.41	0.63
	Dyspnea	392 (78)	148 (74)	240 (79)	0.7 (0.5–1.1)	0.16	345 (58)	147 (55)	198 (60)	0.8 (0.6–1.1)	0.18	0.77
	Expectoration	105 (21)	56 (28)	46 (15)	2.2 (1.4–3.4)	**<0.001**	39 (7)	12 (5)	27 (8)	0.5 (0.3–1.0)	0.069	**<0.001**
	Chest pain	69 (14)	25 (12)	43 (14)	0.9 (0.5–1.5)	0.59	43 (7)	15 (6)	28 (9)	0.6 (0.3–1.2)	0.17	0.47
	Bilateral cracklings	120 (24)	54 (27)	65 (21)	1.4 (0.9–2.0)	0.16	92 (15)	53 (20)	39 (12)	1.8 (1.2–2.9)	**0.0086**	0.34
**Comorbidities**	Renal failure	59 (12)	25 (13)	34 (11)	1.1 (0.6–1.9)	0.68	28 (5)	14 (5)	14 (4)	1.2 (0.6–2.6)	0.6	0.85
	Diabetes	87 (17)	40 (20)	47 (15)	1.4 (0.8–2.2)	0.2	120 (20)	67 (25)	53 (16)	1.7 (1.1–2.6)	**0.0087**	**0.45**
	Chronic lung disease	169 (34)	57 (28)	109 (36)	0.7 (0.5–1.0)	0.075	121 (20)	32 (12)	89 (27)	0.4 (0.2–0.6)	**<0.001**	0.028
	Myocardial infarction	38 (7.6)	17 (8)	21 (7)	1.2 (0.6–2.4)	0.53	42 (7)	19 (7)	23 (7)	1.0 (0.5–1.9)	0.99	0.65
	Cardiac failure	69 (14)	29 (14)	40 (13)	1.1 (0.7–1.9)	0.69	27 (5)	8 (3)	19 (6)	0.5 (0.2–1.1)	0.11	0.11
	Stroke	51 (10)	24 (12)	27 (9)	1.4 (0.8–2.5)	0.27	27 (5)	7 (3)	20 (6)	0.4 (0.2–0.9)	**0.046**	**0.023**
	Metastatic cancer	55 (11)	17 (8)	38 (12)	0.6 (0.3–1.2)	0.15	22 (4)	9 (3)	13 (4)	0.8 (0.3–2.0)	0.69	0.63
	Dementia	43 (8.5)	15 (7)	27 (9)	0.8 (0.4–1.6)	0.57	14 (2)	4 (2)	10 (3)	0.5 (0.1–1.4)	0.22	0.43
	Hemiplegia	6 (1.2)	3 (1)	3 (1)	1.5 (0.3–8.3)	0.61	7 (1)	2 (0.75)	5 (2)	0.5 (0.1–2.3)	0.39	0.33
	System disease	7 (1.4)	1 (0.5)	6 (2)	0.2 (0.0–1.5)	0.2	17 (3)	9 (3)	8 (3)	1.4 (0.5–3.7)	0.51	0.15
	Vascular disease	9 (1.8)	3 (2)	6 (2)	0.8 (0.2–2.9)	0.69	17 (3)	7 (3)	10 (3)	0.8 (0.3–2.2)	0.74	0.89
**Constants**	Respiratory rate (min^-1^)	18 (16–24)	18 (17–25)	18 (16–22)	1.0 (1.0–1.0)	0.79	18 (16–23)	20 (16–25)	18 (16–20)	1.1 (1.0–1.1)	**<0.001**	**<0.001**
	CRP (mg/L)	52 (16–103)	60 (22–116)	42 (11–98)	1.0 (1.0–1.0)	0.97	54 (10–118)	85 (40–146)	20 (4–84)	1.0 (1.0–1.0)	**<0.001**	**0.029**
	NT-proBNP (ng/L)	1796 (521–5034)	1489 (398–6735)	2077 (548–4600)	1.0 (1.0–1.0)	0.16	178 (50–1524)	117 (46–393)	488 (62–2746)	1.0 (1.0–1.0)	**0.0035**	**0.0014**
	Leukocytes (G/L)	9 (7–13)	9 (6–12)	10 (7–13)	0.9 (0.9–1.0)	**<0.001**	8 (6–11)	6 (5–7)	9 (7–12)	0.8 (0.7–0.8)	**<0.001**	**<0.001**
	Lymphocytes (G/L)	1 (1–2)	1 (1–2)	1 (1–2)	1.0 (0.9–1.1)	0.92	1 (1–2)	1 (1–1)	1 (1–2)	0.8 (0.6–1.0)	**0.042**	0.065
	Platelets (G/L)	222 (176–283)	195 (162–260)	239 (183–302)	1.0 (1.0–1.0)	**<0.001**	212 (165–268)	182 (147–222)	240 (191–295)	1.0 (1.0–1.0)	**<0.001**	**0.023**
**Outcome**	ICU admission	26 (5)	10 (5)	16 (5)	0.9 (0.4–2.1)	0.88	77 (13)	65 (24)	12 (4)	8.4 (4.6–16.8)	**<0.001**	**<0.001**
	Intra hospital mortality	15 (3)	6 (3)	9 (3)	1.0 (0.3–2.8)	0.99	76 (13)	52 (19)	24 (7)	3.0 (1.8–5.2)	**<0.001**	0.063

Patients with a negative PCR presented similar characteristics between both periods, except for age–patients were older during the SARS-CoV-2 period (73 [63–85] and 63 [43–72] years, p < 0.001)–and cardiac failure, which was more prevalent during the SARS-CoV-2 period (13% vs. 5.8%, p < 0.002; Table 1). Due to these few but significant differences between the two negative-PCR populations from the two periods, we conducted separate analyses of the factors associated with a positive PCR for any RV (in the RV period) and SARS-CoV-2 (in the SARS-CoV-2 period) in comparison with their own PCR-negative populations.

### Virological findings

During the first study period, 216/508 (43%) patients had a positive mPCR, including 13 dual viral coinfections, leading to the following viral distribution: 68 (31%) rhinovirus, 60 (28%) influenza, 35 (16%) RSV, 30 (14%) human metapneumovirus, 28 (13%) coronaviruses, four (2%) parainfluenza, three (0.1%) adenovirus and one (0.5%) bocavirus. During the second study period, 268/596 (45%) patients had a positive PCR for SARS-CoV-2, and 70 other respiratory viral infections were also identified, including 18 co-infections with SARS-CoV-2. As patients' populations presented some differences across the two periods, patients with a non-SARS-CoV-2 virus were analyzed as SARS-CoV-2-negative patients, while those with a SARS-CoV-2 co-infection were considered as SARS-CoV-2-positive.

### Patients' characteristics associated with the detection of a non-SARS-CoV-2 respiratory virus

The median duration of symptoms was 2 [1–4] days before ED admission. The following characteristics were associated with a positive mPCR in the univariate analysis (Table 1): younger age, presence of fever, cough, and expectorations, and lower white blood cell and platelet counts. These variables were also retrieved in the multivariate analysis, except for lower white blood cell count (Table 2).

**Table 2:** Multivariate analysis for positive PCR for both periods (non-SARS-CoV-2 Respiratory Viruses (RV), and SARS-CoV-2) separately, and for SARS-CoV-2 vs RV in a modeled co-circulation period.

PCR + during RV period (N = 492)*		PCR + during SARS-CoV-2 period (N = 582)*		SARS-CoV-2 vs other respiratory viruses (overall study period)**	
aOR (95%CI)	p	aOR (95%CI)	p	aOR (95%CI)	p
0.89 (0.80–1.00)	0.041			0.80 (0.72–0.89)	<0.001
		2.23 (1.53–3.25)	<0.001	1.64 (1.10–2.44)	0.015
1.70 (1.05–2.75)	0.031			0.13 (0.07–0.26)	<0.001
1.60 (1.08–2.38)	0.02	3.33 (2.25–4.97)	<0.001	3.27 (2.17–4.94)	<0.001
		1.62 (1.03–2.56)	0.038		
		1.72 (1.10–2.70)	0.017		
2.16 (1.43–3.30)	<0.001				
		0.40 (0.24–0.64)	<0.001	0.40 (0.25–0.64)	<0.001
		1.06 (1.02–1.09)	<0.001		
0.69 (0.54–0.85)	<0.001				
0.97 (0.93–1.01)	0.11				

* Number of patients included in this analysis, excluding patients with missing values.

** *i*.*e*. 40 and 45% for non-SARS-CoV-2 viruses and SARS-CoV-2, respectively.

### Clinical and biological characteristics associated with the detection of SARS-CoV-2

The median duration of symptoms was 3 [2–7] days, and the following characteristics were associated with a positive SARS-CoV-2 PCR in the univariate analysis (Table 1): male gender, younger age, fever, chills, myalgia, cough, bilateral cracklings, diabetes, chronic lung disease, history of stroke, higher respiratory rate, CRP, lower NT-proBNP, lower leukocytes and lymphocytes counts and lower platelet count. Regarding comorbidities, chronic lung disease, diabetes, and a stroke history were associated with a positive SARS-CoV-2 PCR. In the multivariate analysis, the following features remained associated with SARS-CoV-2 detection (Table 2): younger age, male gender, fever, chills, myalgia, higher respiratory rate and absence of chronic lung disease. A positive PCR for SARS-CoV-2 was also associated with ICU admission (OR, 8.4; 95% CI, 4.6–16.8; p < 0.001) and with mortality during hospitalization (OR, 3.0; 95% CI, 1.8–5.2; p < 0.001).

### Comparison of human respiratory viruses and characteristics associated with SARS-CoV-2

Under the hypothesis of SARS-CoV-2 co-circulation along with other RVs, variables associated with the virologic diagnosis (SARS-CoV-2 vs other viruses) among positive PCR were as follows (Table 2): male gender (aOR, 1.64; 95% CI, 1.10–1.44; p = 0.015), absence of chronic lung disease (0.40, 0.25–0.64, <0.001), age (0.80 per 10 years, 0.72–0.89, <0.001), presence of fever (3.27, 2.17–4.94, <0.001) and absence of expectorations (0.13, 0.07–0.26, <0.001). A clinical score based on this multivariate analysis was computed using integer coefficients based on OR logarithms (S2 Table), ranging from -6 to +5. Under the baseline scenario conditions, corresponding to the overall period of the study with a prevalence of 43% of positive PCR and distribution of 56% and 44% of SARS-CoV-2 and non-SARS-CoV-2 viruses, respectively, among positive PCR, our score has an AUC of 0.81 (95% CI 0.77–0.85; S1 Fig). Furthermore, when using a score greater than 1 to predict SARS-CoV-2 among all viral infections, we observed sensitivity and a specificity of 83% and 65%, respectively. To note, with a score greater than or equal to 1, the sensitivity and specificity were at 91% and 52%, respectively.

To test our observations' robustness in case of variable prevalence of SARS-CoV-2 among all RVs, two other scenarios were added, assuming the same global positivity rate of 43% with a dominant or a limited SARS-CoV-2 distribution circulation among positive PCR at 75% and 25%, respectively. The following factors were associated with COVID-19 diagnosis in all scenarios: younger age, male gender, fever, myalgia, dyspnea, absence of expectoration, and absence of chronic lung disease (Cf. S1 Table). The positive predictive value (PPV) and the negative predictive value (NPV) with a score greater than one were at 75 and 76% with the viral distribution observed in our study. Under the dominant and the limited SARS-CoV-2 distribution scenarios (i.e., 75% and 25% probability of SARS-CoV-2 among positive patients), the PPVs were at 92% and 57%, respectively, and the NPVs were at 45 and 88%, respectively.

## Discussion

In this large monocentric prospective cohort study, including 508 and 596 consecutive ILI patients attending the ED during the RV and SARS-CoV-2 periods, respectively, we identified several clinical features associated with positive viral PCR. This allowed us to identify the highest COVID-19 suspicions among all respiratory viral infections.

The ability to distinguish COVID-19 from other RVs will become an increasingly important issue in northern-hemisphere countries as circulation of SARS-CoV-2 could be expected to continue during the upcoming year and the next epidemic of winter-associated viruses [5]. The importance of such co-circulation is challenging to predict. Few countries in the southern hemisphere are currently describing high co-circulation of other RVs with SARS-CoV-2. However, several countries at the beginning of the SARS-CoV-2 outbreak in Northern America or Europe reported such co-circulation [6, 7].

The features observed in our cohort for COVID-19 patients are in line with previous reports [14]. Most of these clinical features are also associated with other respiratory viral infections observed in our study and previous works [15, 16]. By analyzing the comparative strength of these clinical features associations with SARS-CoV-2 positivity in relation to other RVs, we were able to define a limited set of markers associated with a higher risk of being infected by SARS-CoV-2: being male, of a younger age, with feverishness and in the absence of expectoration is predictive of a SARS-CoV-2 infection while having chronic lung diseases is predictive of non-SARS-CoV-2 RVs. Our results were confirmed under several SARS-CoV-2 prevalence conditions and are in line with the only other study available to date comparing features associated with SARS-CoV-2 to those associated with other respiratory viral infections. Cardiovascular diseases also seem to be associated to SARS-CoV-2 diagnosis, in our model under the different scenarios. This observation is also in line with previous publications that have considered cardiovascular diseases as a biomarker for an increased risk of COVID-19 infection and poor outcomes with a case fatality rate of 10.5% [17, 18].

A small French cohort has also described anosmia, dysgeusia, diarrhea, frontal headache, and bilateral crackling sounds more frequently associated with COVID-19 than other RVs [19]. Our results confirm the findings of this smaller retrospective work on a large prospective cohort, except for diarrhea, which was not associated with COVID-19 diagnosis in our population. In both this previous work and our study, no biological findings upon ED admission allowed for discrimination between SARS-CoV-2 and non-SARS-CoV-2 respiratory infections. An interesting point highlighted in our work is the impact of SARS-CoV-2 and the other RVs relative prevalence on the clinical scoring with PPVs ranging from 57 to 92% and NPVs ranging from 45 to 88% depending on the proportion of SARS-CoV-2 among RVs. As that relative prevalence cannot be predicted and will probably evolve during the RV epidemic period, any clinical scoring of COVID-19 suspicion will have to be monitored in real time and will not eliminate the need for rapid molecular assays.

Our study presents several limitations. First, it is a monocentric study, and the RV period was designed before the emergence of SARS-CoV-2 for analysing other RV features. Therefore, a few key characteristics, such as diarrhea, anosmia, and ageusia, or procalcitonin, which was initially not known to be associated with COVID-19, could not be included in the present work. Their collection could help in future works to improve the clinical scoring of COVID-19 suspicions. As the French COVID-19 initial outbreak began at the end of the winter season, a few other RVs were identified during the SARS-CoV-2 period, alone or in association with SARS-CoV-2. The small numbers of co-infections observed in our work did not allow for an individual assessment of their clinical and biological presentations. Finally, the proposed clinical score requires an external validation before being used for patient management. Moreover, the identified features may evolve in the upcoming years with the ongoing immunization of the general population. Surveillance of the evolution of the disease will, therefore, be required in the future.

In conclusion, symptoms associated with SARS-CoV-2 and other respiratory viral infections are frequently shared [8]. This poses potential challenges in patient management in case of a large co-circulation of all these viruses, as expected in the upcoming months in most northern countries. Despite this overlap, COVID-19 patients present several clinical characteristics less frequently identified among those infected by other RVs such as influenza. Based on this data, we developed a clinical tool to aid in screening SARS-CoV-2 infection among all viral respiratory infections. These observations were confirmed in various ratios of SARS-CoV-2 and other RVs. As having an efficient, reliable, and rapid patient's triage system upon ED entrance, clinical scoring could be a tempting and useful tool depending on one's local environment and constraints, mainly due to the considerable PCR turnaround time or low single room availability either in the ED or in the hospital. However, this tool needs to be prospectively evaluated before any potential use, and, as we demonstrated, the performances may be strongly impacted by the relative prevalence of SARS-CoV-2 and other RVs. As this relative prevalence is impossible to predict soon, no clinical scoring will waive the need for molecular assays. Therefore, on the eve of the next respiratory viruses epidemic period, our primary efforts should still focus on having a large availability, accessibility, and optimized use of rapid molecular testing.

## Supporting information

S1 Fig ROC curves of the multivariate model of RV period (left, N = 492), SARS-CoV-2 period (middle, N = 582) and of the clinical score for SARS-Cov-2 discrimination (right, N = 463). (DOCX)

S1 Table Factors associated with a positive PCR for SARS-CoV-2 given that the PCR is positive to at least one respiratory virus (modeling under different scenarios, univariate analysis; baseline scenario corresponds to the union of both samples, low and high COVID scenario). (DOCX)

S2 Table The clinical score for SARS-CoV-2 suspicion w.r.t. other respiratory viruses. A score strictly greater than one has a sensitivity of 83% and a specificity of 65% of presenting a SARS-CoV-2 instead of any other respiratory viruses in case of positive PCR.
(DOCX)
