# Correction: Characteristics associated with COVID-19 or other respiratory viruses’ infections at a single-center emergency department

**DOI:** 10.1371/journal.pone.0246458

**Published:** 2021-01-27

**Authors:** Donia Bouzid, Jimmy Mullaert, Quentin Le Hingrat, Odile Laurent, Xavier Duval, Xavier Lescure, Jean-François Timsit, Diane Descamps, Philippe Montravers, Christophe Choquet, Jean-Christophe Lucet, Enrique Casalino, Benoit Visseaux, ED influenza study group

In Table 2, the first column containing the variable names is missing. Please see the correct Table 2 here.

**Table 2:** Multivariate analysis for positive PCR for both periods (non-SARS-CoV-2 Respiratory Viruses (RV), and SARS-CoV-2) separately, and for SARS-CoV-2 vs RV in a modeled co-circulation period.

	PCR + during RV period (N = 492)*		PCR + during SARS-CoV-2 period (N = 582)*		SARS-CoV-2 vs other respiratory viruses (overall study period)**	
	aOR (95%CI)	p	aOR (95%CI)	p	aOR (95%CI)	p
Age (per 10 years)	0.89 (0.80–1.00)	0.041			0.80 (0.72–0.89)	<0.001
Male gender			2.23 (1.53–3.25)	<0.001	1.64 (1.10–2.44)	0.015
Expectoration	1.70 (1.05–2.75)	0.031			0.13 (0.07–0.26)	<0.001
Fever	1.60 (1.08–2.38)	0.02	3.33 (2.25–4.97)	<0.001	3.27 (2.17–4.94)	<0.001
Chills			1.62 (1.03–2.56)	0.038		
Myalgia			1.72 (1.10–2.70)	0.017		
Cough	2.16 (1.43–3.30)	<0.001				
Diabetes						
Chronic lung disease			0.40 (0.24–0.64)	<0.001	0.40 (0.25–0.64)	<0.001
Respiratory rate (min^-1^)			1.06 (1.02–1.09)	<0.001		
Platelets (per 100G/L)	0.69 (0.54–0.85)	<0.001				
Leucocytes (per G/L)	0.97 (0.93–1.01)	0.11				

* Number of patients included in this analysis, excluding patients with missing values

** *i*.*e*. 40 and 45% for non-SARS-CoV-2 viruses and SARS-CoV-2, respectively
