# Supplementary material for: Characteristics associated with COVID-19 or other respiratory viruses’ infections at a single-center emergency department
Source: PLoS One. 2020 Dec 3;15(12):e0243261. doi: 10.1371/journal.pone.0243261 (PMC7714208; doi:10.1371/journal.pone.0243261)
Supplement: S1 Fig — (DOCX) [file pone.0243261.s001.docx]

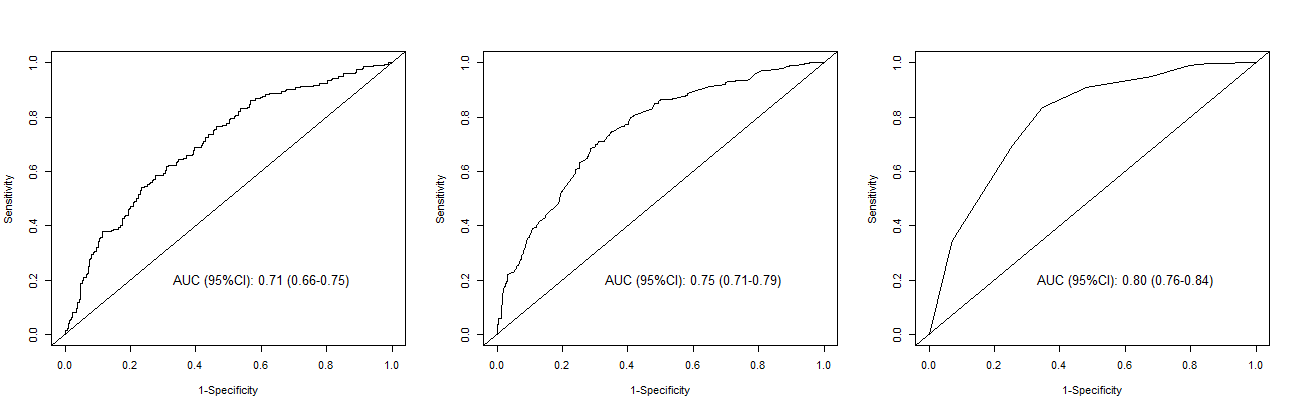


**S1 Fig.** ROC curves of the multivariate model of RV period (left, N=492), SARS-CoV-2 period (middle, N=582) and of the clinical score for SARS-Cov-2 discrimination (right, N=463).
