# Supplementary material for: Characteristics associated with COVID-19 or other respiratory viruses’ infections at a single-center emergency department
Source: PLoS One. 2020 Dec 3;15(12):e0243261. doi: 10.1371/journal.pone.0243261 (PMC7714208; doi:10.1371/journal.pone.0243261)
Supplement: S1 Table — (DOCX) [file pone.0243261.s002.docx]

| **Co-circulation scenario** |  | **Low COVID scenario** positive PCR 43% SARS-CoV-2 proportion 25% | | **Baseline scenario** positive PCR 43%  SARS-CoV-2proportion 56% | | **High COVID scenario** positive PCR 43% SARS-CoV-2proportion 75% | |
| --- | --- | --- | --- | --- | --- | --- | --- |
|  |  | OR (95%CI) | p | OR (95%CI) | p | OR (95%CI) | p |
| **General** | Sex: male | 2.29 (1.47-3.58) | **0.00027** | 1.85 (1.27-2.70) | **0.0015** | 1.63 (1.06-2.50) | **0.025** |
|  | Age | 0.97 (0.96-0.98) | **1.9e-07** | 0.97 (0.96-0.98) | **1,00E-06** | 0.98 (0.97-0.99) | **0.00014** |
|  | Symptoms duration (days) | 1.05 (1.01-1.09) | **0.01** | 1.05 (1.01-1.10) | **0.0098** | 1.06 (1.01-1.11) | **0.025** |
| **Symptoms** | Fever | 6.60 (4.05-10.75) | **3.6e-14** | 3.88 (2.61-5.75) | **1.6e-11** | 2.83 (1.83-4.38) | **2.8e-06** |
|  | Hypothermia | 0.54 (0.15-2.01) | 0.36 | 0.71 (0.24-2.07) | 0.53 | 0.82 (0.24-2.77) | 0.75 |
|  | Chills | 2.13 (1.29-3.53) | **0.0033** | 1.69 (1.06-2.70) | **0.027** | 1.50 (0.87-2.59) | 0.14 |
|  | Sweats | 1.48 (0.73-3.00) | 0.28 | 1.31 (0.69-2.49) | 0.41 | 1.22 (0.58-2.58) | 0.6 |
|  | Headaches | 3.39 (1.71-6.73) | **0.00048** | 3.11 (1.55-6.25) | **0.0014** | 2.97 (1.27-6.98) | **0.012** |
|  | Myalgia | 3.22 (1.95-5.34) | **5.4e-06** | 2.43 (1.50-3.94) | **0.00031** | 2.09 (1.18-3.72) | **0.012** |
|  | Malaise | 0.60 (0.24-1.51) | 0.28 | 0.68 (0.32-1.45) | 0.32 | 0.73 (0.31-1.70) | 0.46 |
|  | Cough | 1.49 (0.94-2.38) | 0.093 | 0.98 (0.65-1.47) | 0.93 | 0.78 (0.49-1.24) | 0.3 |
|  | Sore Throat | 1.59 (0.69-3.66) | 0.27 | 1.28 (0.59-2.74) | 0.53 | 1.12 (0.46-2.71) | 0.8 |
|  | Dyspnea | 0.36 (0.23-0.56) | **5,00E-06** | 0.41 (0.28-0.62) | **2,00E-05** | 0.45 (0.28-0.72) | **0.00095** |
|  | Expectoration | 0.13 (0.05-0.30) | **4.1e-06** | 0.12 (0.06-0.22) | **1.7e-11** | 0.11 (0.06-0.20) | **3,00E-12** |
|  | Chest pain | 0.35 (0.15-0.80) | **0.013** | 0.41 (0.21-0.78) | **0.0064** | 0.44 (0.22-0.89) | **0.022** |
|  | Bilateral cracklings sounds | 0.85 (0.50-1.43) | 0.54 | 0.66 (0.42-1.04) | 0.07 | 0.58 (0.35-0.95) | **0.031** |
| **Comorbidities** | Renal failure | 0.42 (0.17-1.02) | 0.056 | 0.39 (0.19-0.78) | **0.0075** | 0.37 (0.18-0.77) | **0.0078** |
|  | Diabetes | 1.69 (1.02-2.79) | **0.042** | 1.34 (0.85-2.11) | 0.21 | 1.18 (0.70-1.99) | 0.54 |
|  | Chronic respiratory failure | 0.24 (0.13-0.43) | **1.7e-06** | 0.33 (0.21-0.52) | **2.1e-06** | 0.41 (0.25-0.66) | **0.00032** |
|  | Myocardial infarction | 0.88 (0.39-1.97) | 0.75 | 0.82 (0.41-1.64) | 0.58 | 0.79 (0.36-1.73) | 0.56 |
|  | Heart failure | 0.16 (0.06-0.47) | **0.00086** | 0.18 (0.09-0.39) | **1.2e-05** | 0.20 (0.09-0.42) | **2.7e-05** |
|  | Stroke | 0.18 (0.06-0.56) | **0.0031** | 0.20 (0.09-0.44) | **8.3e-05** | 0.21 (0.09-0.47) | **0.00015** |
|  | Metastatic cancer | 0.32 (0.11-0.90) | **0.032** | 0.38 (0.17-0.84) | **0.017** | 0.41 (0.18-0.97) | **0.041** |
|  | Dementia | 0.15 (0.03-0.65) | **0.011** | 0.18 (0.06-0.52) | **0.0014** | 0.21 (0.08-0.58) | **0.0027** |
|  | Hemiplegia | 0.48 (0.05-4.43) | 0.51 | 0.50 (0.09-2.84) | 0.43 | 0.51 (0.08-3.33) | 0.48 |
|  | System disease | 5.15 (1.14-23.26) | **0.033** | 6.94 (1.11-43.40) | **0.038** | 7.83 (0.67-91.70) | 0.1 |
|  | Vascular disease | 1.56 (0.39-6.24) | 0.53 | 1.77 (0.47-6.66) | 0.4 | 1.89 (0.38-9.40) | 0.44 |
| **Constants** | Temperature (C°) | 1.94 (1.60-2.36) | **1.7e-11** | 1.64 (1.40-1.91) | **4.1e-10** | 1.49 (1.26-1.75) | **1.9e-06** |
|  | Respiratory rate (min^-1^) | 1.03 (1.00-1.06) | 0.058 | 1.01 (0.99-1.04) | 0.3 | 1.01 (0.98-1.04) | 0.69 |
|  | C Reactive Protein (mg/L) | 1.00 (1.00-1.01) | 0.14 | 1.00 (1.00-1.01) | 0.41 | 1.00 (1.00-1.01) | 0.65 |
|  | NT-proBNP (ng/L) | 1.00 (1.00-1.00) | **0.00046** | 1.00 (1.00-1.00) | **8.8e-07** | 1.00 (1.00-1.00) | **3.4e-07** |
|  | Leukocytes (G/L) | 0.78 (0.62-0.98) | **0.031** | 0.84 (0.79-0.90) | **4.1e-07** | 0.88 (0.84-0.93) | **5.1e-06** |
|  | Lymphocytes (G/L) | 0.70 (0.00- NA) | 0.98 | 0.87 (0.62-1.21) | 0.4 | 0.96 (0.83-1.10) | 0.56 |
|  | Platelets (G/L) | 0.99 (0.99-1.00) | **6.4e-06** | 1.00 (0.99-1.00) | **0.0012** | 1.00 (1.00-1.00) | 0.088 |
| **Outcome** | ICU admission | 10.73 (5.58-20.63) | **1.1e-12** | 6.17 (3.04-12.51) | **4.6e-07** | 4.91 (2.04-11.83) | **4,00E-04** |
|  | Intra hospital mortality | 10.75 (4.93-23.41) | **2.3e-09** | 7.89 (3.28-18.98) | **4.0e-06** | 6.81 (2.25-20.61) | **0.00069** |

**S1 Table.** Factors associated with a positive PCR for SARS-CoV-2 given that the PCR is positive to at least one respiratory virus (modeling under different scenarios, univariate analysis; baseline scenario corresponds to the union of both samples, low and high COVID scenario )

OR: odd ratio for SARS-CoV-2 detection among positive PCR
