# Supplementary material for: Characteristics associated with COVID-19 or other respiratory viruses’ infections at a single-center emergency department
Source: PLoS One. 2020 Dec 3;15(12):e0243261. doi: 10.1371/journal.pone.0243261 (PMC7714208; doi:10.1371/journal.pone.0243261)
Supplement: S2 Table — A score strictly greater than one has a sensitivity of 83% and a specificity of 65% of presenting a SARS-CoV-2 instead of any other respiratory viruses in case of positive PCR. (DOCX) [file pone.0243261.s003.docx]

| Variable | score |
| --- | --- |
| Fever | 2 |
| Age< 60 | 2 |
| Sex (male) | 1 |
| Chronic lung disease | -2 |
| Expectoration | -4 |

**S2 Table.** The clinical score for SARS-CoV-2 suspicion w.r.t. other respiratory viruses. A score strictly greater than one has a sensitivity of 83% and a specificity of 65% of presenting a SARS-CoV-2 instead of any other respiratory viruses in case of positive PCR.
